# Risk factors of morbidity among children under age five in Ethiopia

**DOI:** 10.1186/s12889-019-7273-4

**Published:** 2019-07-15

**Authors:** Kasahun Takele, Temesgen Zewotir, Denis Ndanguza

**Affiliations:** 1 African Center of Excellence in Data Science, University of Rwanda, Kigali, Rwanda; 2 School of Mathematics, Statistics and Computer Sciences, University of KwaZulu-Natal, Durban, South Africa; 3 College of Science and Technology, University of Rwanda, Kigali, Rwanda

**Keywords:** EDHS, GEE, ALR, Diarrhea, Fever

## Abstract

**Background:**

Childhood morbidities are a major cause of mortality of children in the developing countries particularly in Ethiopia. Regardless of the noticeable improvement in the reduction of under-five death in Ethiopia, childhood diarrhea and fever are still the leading cause of death. In Ethiopia, the burden of child mortality is alarming and calls for determined efforts in combating such health problems. Therefore, this study aimed to investigate the risk factors for childhood morbidity specifically for diarrhea and fever.

**Methods:**

To gain insight into children’s health issues, the 2016 Ethiopian Demographic and Health Survey data were used. Among the marginal models, alternating logistic regression that is an extension of the generalized estimating equation model was used to investigate the risk factors of childhood morbidity explicitly for diarrhea and fever.

**Results:**

The results show that the child’s sex, child’s age, anemia level, husband education level, mother’s work status, mother’s marital status, breastfeeding status and region are all chosen significant risk factors related with childhood diarrhea disease and fever disease.

**Conclusion:**

The study indicated that male children, 0–11 months aged children, 12–23 months aged children, anemic children, husband with a lower education, mothers paid employment, non-breastfed children, regions of Amhara, Afar, Dire Dawa, Benishangul, Gambela, Oromia, SNNPR, Somali and Tigray were significantly associated with higher odds of morbidity in Ethiopia. Therefore, there is a need for children morbidity interventions intended to improve child health outcomes in the country.

## Background

Childhood diseases are among the most serious health issues facing developing countries. In 2016, globally acute respiratory infections, diarrhea and malaria were the leading causes of children under five death particularly 8% of deaths among children under age five caused by diarrhea [1]. According to the UNICEF report in 2016, approximately 5.6 million children under age of five die every year, which is decreased from over 12 million in 1990. Most of these children (80%) are from sub-Saharan African and Southern Asia. Regardless of the considerable improvement in reducing childhood death, childhood survival persists a critical concern. Globally, about 75% of the under-five deaths are still caused by a handful of conditions like diarrhea, fever, cough and malaria. Furthermore, it is insupportable that 15,000 children die daily, commonly from preventable and treatable diseases such as diarrhea, malaria and fever [2]. Some of previous studies revealed that as diarrhea and fever are among the prevalent diseases that contributes to the burden of childhood morbidity and mortality [3]. Separately from its serious influence on child mortality, diarrhea and fever can result in long-term health effects, including depletion of immune strength, malnutrition and making children susceptible to other diseases [4, 5].

Numerous investigations in Africa revealed that infectious diseases as the leading causes of under age of five children death [6–9]. In Ethiopia, childhood morbidity and mortality remain high due to the burden associated with highly prevalent diseases such as diarrhea, fever, cough, malaria and HIV-AIDS. For instance, diarrhea contributes to more than one in every ten (13%) child deaths in Ethiopia [10]. Furthermore, according to the 2016 Ethiopia Demographic and Health Survey report, 12% of under-five children had a diarrheal episode and 14% of under-five children had a fever episode in the 2 weeks prior the survey [11]. This has encouraged medical researchers and statisticians to gain insight into this health problem with the view to developing strategies to combat it.

Likewise, some of studies have investigated the association between diseases and socioeconomic, demographic, environmental, and individual risk factors in Ethiopia and many other sub-Saharan African countries. Most of these investigations used logistic regression to identify the significant risk factors of diseases that associated to children morbidity [6, 12–15]. In addition, other articles focused only on diarrhea, using simple statistical analyses [12, 16, 17]. However, these previous studies on children diseases ignored association between children from the same household. As results, inference associated with the estimated parameters may not be correct means that the standard errors may be too small resulting in *p*-values that are too small and confidence intervals that are too narrow [18]. Furthermore, little is known and much work remains to be done to develop a better accepting approach that allows us to investigate childhood morbidities in a more accurate way.

The main objective of this work was to examine the effect of the socioeconomic, demographic, household and spatial characteristics related to children morbidity using the 2016 Ethiopia Demographic and Health Survey data. Identification of determinants of children diseases tends to assist to guide strategic planning, address strength and weakness of current policy, prioritize future research questions and interventions for addressing children morbidity and mortality problem in Ethiopia.

## Methods

### Data source

Ethiopia is a landlocked country located in East Africa, which categorized as sub-Saharan Africa. In this paper, we used the data set available from the 2016 Ethiopian DHS. This survey is the fourth cross-sectional investigation administered at household level. A stratified two-stage cluster sampling procedure was used. Enumeration areas (EA) were the sampling units for the first stage and list of households in EA were the sampling unit in the second stage. The sample included 645 enumeration areas among 202 in urban areas and 443 in rural areas. The survey included questions designed to explore sociodemographic, socioeconomic, child health, maternal and environmental conditions at household level. The data was collected from women interviewees aged 15–49 years. In the survey, the health status of each interviewee’s children aged less than 60 months in the 2016 survey was assessed by asking the interviewee ‘Has your child had diarrhea, and fever in the last two weeks?’ The occurrence of diarrhea and fever categorized as “Yes’ or “No.’ Overall, data on 8,742 ‘young’ children was collected in the survey [11]. Table 1 provides information on categorical socioeconomic and demographic covariates, their categories, frequencies and association with diarrhea and fever. Childhood diarrhea defined as the frequent (three or more times per day) loss of liquid stools within 2 weeks period preceding to survey. Fever is an abnormally high body temperature, accompanied by shivering, headache, and restlessness [19]. The details on sampling methodology used in the survey can be found in [11].

**Table 1:** Distribution of childhood morbidity and its associated selected risk factors

Covariates	Diarrhea recently (N and %)			Fever recently (N and %)		
	No	Yes	*p*-value	No	Yes	*p*-value
Current age of child						
0–11 months	1604(86.1)	260(13.9)	0.000	299 (16.0)	299 (16.0)	0.000
12–23 months	1449(82.1)	316 (17.9)		1421 (80.5)	344 (19.5)	
24–59 months	4672(91.4)	441 (8.6)		4499 (88.0)	614 (12.0)	
Sex of child						
Male	3912 (87.8)	542 (12.2)	0.012	3806 (85.5)	648 (14.5)	0.654
Female	3813 (88.9)	475 (11.1)		3679 (85.8)	609 (14.2)	
Anemia level						
Anemic	7517 (88.2)	1003 (11.8)	0.012	7283 (85.5)	1237 (14.5)	0.021
Not anemic	208 (93.7)	14 (6.3)		202 (91.0)	20 (9.0)	
Husband education level						
No education	3995 (89.7)	457 (10.3)	0.000	3858 (86.7)	594 (13.3)	0.000
Primary	2389 (86.7)	367 (13.3)		2298 (83.4)	458 (16.6)	
Secondary	776 (86.5)	121 (13.5)		763 (85.1)	134 (14.9)	
Higher	565 (88.7)	72 (11.3)		566 (88.9)	71 (11.1)	
Currently working						
No	5609 (88.9)	703 (11.1)	0.020	5453 (86.4)	859 (13.6)	0.001
Yes	2116 (87.1)	314 (12.9)		2032 (83.6)	398 (16.4)	
Marital status						
Never in union	51 (91.1)	5 (8.9)	0.696	42 (75.0)	14 (25.0)	0.002
Married	7300 (88.4)	960 (11.6)		7089 (85.8)	1171 (14.2)	
Widowed	94 (90.4)	10 (9.6)		95 (91.3)	9 (8.7)	
Separated	280 (87.0)	42 (13.0)		259 (80.4)	63 (19.6)	
Breastfeeding status						
No	2589 (89.7)	298 (10.3)	0.007	2470 (85.6)	417 (14.4)	0.015
Yes	5136 (87.7)	719(12.3)		5015 (85.7)	840 (14.3)	

### Marginal models

Generalized Estimating Equation (GEE) and Alternating Logistic Regression (ALR) were used to model childhood morbidity, and compare the odds of a child being had diarrhea and /or fever given the various risk factors considered. Within these GEE and ALR models framework the households were considered to be clustered and not independent within each household. The response variables were the occurrence of diarrhea and/or fever (Yi: binominal variable, i.e., yes or no), and for this study we considered that the event occurred if the child had diarrhea and/or fever in the last 2 weeks of the survey. Consequently, the response variable was coded with 1 for child had diarrhea and/or fever and with 0 for child had no diarrhea and/or fever. The link function between the mean value *Y*_*i*_ and the model covariates considered for GEE and working correlation structure is defined by:

1 $$ g\left({\omega}_i\right)= logit\left({\omega}_j\right)={x_i}^{\prime}\beta $$

Where *g*(*ω*_*i*_) is logit link function, *x*_*i*_ is *n*_*i*_ ∗ *x*_*i*_ dimensional vector of known covariates, *β* = (1*xp*) dimensional vector of unknown parameters, *E*(*Y*_*i*_) = *ω*_*i*_ is expected value of the response *Y*_*i*_ in the cluster i which is binomially distributed as *y*_*i*_~*bin*(*n*_*i*_, *ω*_*i*_). In addition, GEE is non-likelihood method that captures the association within households in terms of marginal correlations [18]. With this GEE model, the correlation structure of the data within each household was assumed to be of the independence, unstructured, exchangeable, and first order auto-regressive [20]. The parameter ***β*** are estimated by quasi-likelihood.

However, when the cluster sizes become larger, the simultaneous estimation of marginal mean and dependence structure can become computationally prohibitive using GEE. As a result, alternating logistic regression which measures pairwise association of two observations in the same household and follow the precision estimates for both the regression (***β***) and the association (***α***) parameters considered [18, 21]. Furthermore, unlike GEE, no working assumptions about the third order and fourth-order odds ratios are required. Alternating logistic regression measures the association using the odds ratio, which is interpretable and more applicable for binary data [18, 21]. Let *γ*_*ijk*_ be the log odds ratio between outcomes *y*_*ij*_ and *y*_*ik*_, let *μ*_*ij*_ = *p*(*y*_*ij* = 1_) and *ν*_*ijk*_ = *p*(*y*_*ij*_ = 1, *y*_*ik*_ = 1), then the association of the two responses is defined as [22]:

2 $$ logit\ p\left({Y}_{ij}=1/{Y}_{ik}={y}_{ik}\right)={\gamma}_{ij k}{Y}_{ik}+\mathit{\log}\left(\frac{\mu_{ij}-{\nu}_{ij k}}{1-{\mu}_{ij}-{\mu}_{ik}+{\mu}_{ij k}}\right) $$

Similar to GEE, the parameter ***β*** are estimated by quasi-likelihood. Descriptive statistics and chi-squared tests were carried out to identify associations between risk factors and outcome variables (diarrhea and fever) using version 21 of SPSS. *P*-values of less than 0.05 were considered statistically significant. Generalized estimating equation and alternating logistic regression models fit for this study were carried out with the SAS 9.4 version.

### Model selection

The purpose of building a statistical model is to find an optimal model characterized by principles of generalizability, goodness-of-fit and parsimony based on model selection criteria. In this study, three working correlation assumptions exchangeable, first-order autoregressive, AR (1), and independence were used to select the best model with minimum qausi information criteria (QIC) and to select a working correlation structure [23]. In order to select important covariates related to diarrhea and fever backward selection method was used. With this method, we started with a full model containing all main effects and interactions to a more parsimonious model.

## Results

### Exploratory data analysis

**Fig. 1:**
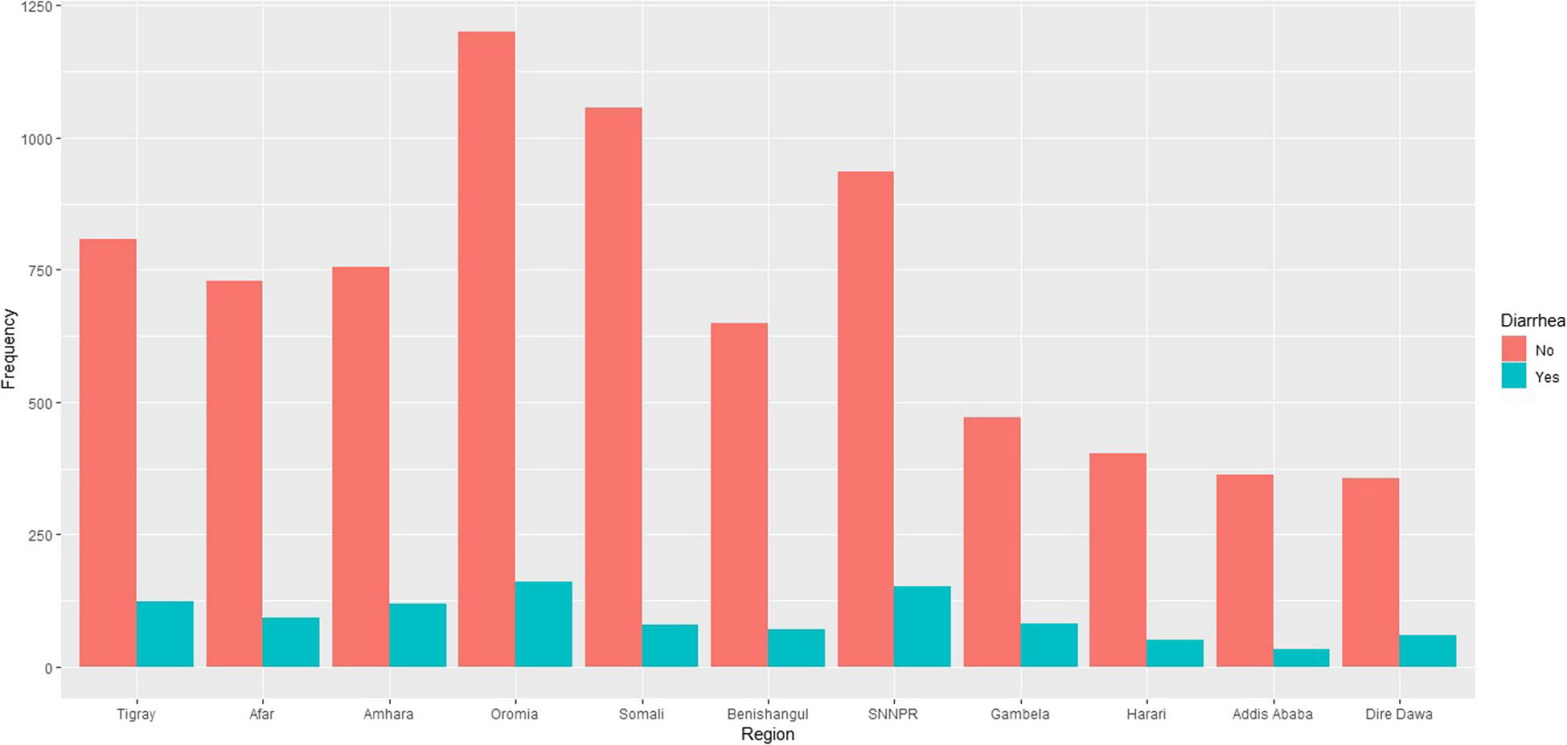
Bar chart of childhood diarrhea morbidity by region

**Fig. 2:**
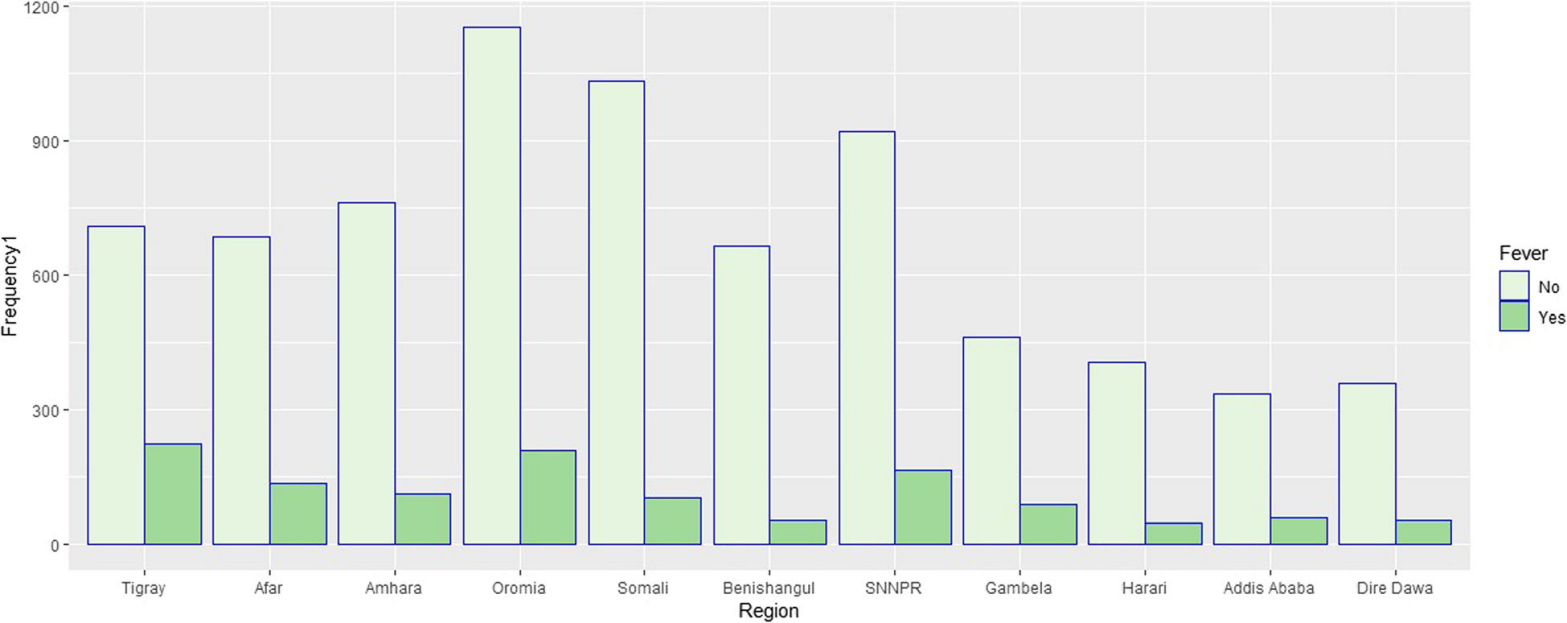
Bar chart of childhood fever morbidity by region

Figure 1 shows the prevalence of diarrhea by region in Ethiopia. It reveals that diarrhea disease (Yes) of children in the country is highly prevalent in Oromia, SNNPR and Tigray followed by Amhara, Afar, Gambela and Somali regions compared to Addis Ababa city. Likewise, the bar chart of Fig. 2 shows that fever disease (Yes) of children in the country is highly prevalent in Oromia, SNNPR and Tigray followed by Afar region compared to Addis Ababa city.

Besides, the results of cross-tabulation analysis for children morbidity are summarized in Table 1. Cross-tabulation analysis indicates that current age of child, anemia level, husband education level and breastfeeding status associated with childhood diarrheal disease and fever disease at a 5% level of significance. Similarly, sex of child and marital status associated with childhood diarrheal disease and fever disease at a 5% level of significance, respectively. Table 1 indicates that higher prevalence of both diseases among children of age group between 12 to 23 months, 17.9% diarrhea and 19.5% fever (diarrhea, *p-*value < 0.000, fever, *p-*value < 0.000).

Moreover, from Table 1, the male children are more likely to have diarrhea compared to female counterpart 12.2% (*p-*value =0.012). The children from mother paid employment are indicates higher prevalence rate of diarrhea and fever diseases than children from mother unemployed (12.9%, *p*-value = 0.020, 16.4%, *p*-value< 0.001) respectively. From the same table, it is indicated that anemic children are more vulnerable to diarrhea and fever 11.8 and 14.5% respectively than non-anemic children (diarrhea, *p-*value =0.012, fever, *p-*value < 0.021). Furthermore, breastfeeding is associated with diarrheal disease and fever disease (diarrhea, *p-*value < 0.000, fever, *p-*value < 0.001). Table 1 shows that non-breastfed children have a lower rate of diarrhea. In addition, breastfed children have a lower rate of fever compared to non-breastfed children (14.3 vs. 14.4%). The education level of the father is highly associated with childhood morbidity (diarrhea, *p-*value < 0.000, fever, *p-*value < 0.000).

#### Results from marginal models

Model with diarrhea as outcome and child sex, child age, anemia, husband education level, mother’s work status and region as predictors were found to be the most parsimonious model. This model has the smallest QIC value for exchange, independence and unstructured working correlation structures. Lastly, exchangeable working correlation assumption was found to be more plausible than the other working correlation assumptions based on comparison of empirical and model based standard errors for the parameter estimates since the two standard errors were very close. Finally, using selected predictors, alternative logistic regression model which provides information about pairwise association of observations among two households within the same cluster was fitted. For diarrhea as outcome variable, the QIC values 6126.9290 and 6126.8773 for GEE and ALR models respectively, with parameter estimates, their corresponding empirically corrected standard errors and *p*-values presented in Table 2. The QIC for GEE and ALR are almost equal. However, empirically corrected standard errors under ALR are somewhat less than those of GEE and the small differences in parameter estimates attributed to the fact that ALR considers the association, but GEE treats the association as a nuisance parameter.

**Table 2:** Parameter estimates and empirical standard errors of GEE and ALR models for Diarrhea

Marginal Model	GEE			ALR	
Effect	par	Est(s.e)	*p*-value	Est(s.e)	*P*-value
Intercept	*β*_0_	−3.3256(0.3445)	<.0001	−3.3279(0.3199)	<.0001
Current age of child (24–59 months = ref)					
0–11 months	*β*_1_	0.5518(0.0843)	<.0001	0.5529(0.0840)	<.0001
12–23 months	*β*_2_	0.8384(0.0801)	<.0001	0.8402(0.0780)	<.0001
Sex of child (Male = ref)					
Female	*β*_3_	−0.1327 (0.0638)	0.0376	−0.1332(0.0637)	0.0366
Anemia level (not anemic = ref.)					
Anemic	*β*_4_	0.5489(0.2641)	0.0377	0.5507(0.2639)	0.0369
Husband education level (No education = ref)					
Higher	*β*_5_	0.0556(0.1454)	0.7019	0.0564(0.1451)	0.6972
Primary	*β*_6_	0.2434(0.0828)	0.0033	−0.2440(0.0827)	0.0032
Secondary	*β*_7_	0.2894(0.1165)	0.0130	−0.2905(0.1163)	0.0125
Currently working(Yes = ref)					
No	*β*_8_	−0.1980(0.0763)	0.0095	−0.1979(0.0762)	0.0094
Region (Addis Ababa = ref)					
Afar	*β*_9_	0.5600(0.2251)	0.0129	0.5610(0.2249)	0.0126
Amhara	*β*_10_	0.7600(0.2136)	0.0004	0.7617(0.2134)	0.0004
Benishangul	*β*_11_	0.2779(0.2267)	0.2204	0.2779(0.2263)	0.2195
Dire Dawa	*β*_12_	0.7616(0.2256)	0.0007	0.7629(0.2254)	0.0007
Gambela	*β*_13_	0.7260(0.2221)	0.0011	0.7280(0.2218)	0.0010
Harari	*β*_14_	0.4263(0.2425)	0.0788	0.4293(0.2420)	0.0761
Oromia	*β*_15_	0.5142(0.2086)	0.0137	0.5144(0.2084)	0.0136
SNNPR	*β*_16_	0.6846(0.2099)	0.0011	0.6860(0.2096)	0.0011
Somali	*β*_17_	0.0310(0.2226)	0.8894	0.0318(0.2223)	0.8863
Tigray	*β*_18_	0.6167(0.2141)	0.0040	0.6193(0.2136)	0.0037
Correlation	ρ	−0.003887364			
Alpha	α			−0.0530(0.0297)	0.0035
QIC		6126.9290		6126.8773	

As a result, ALR model was the better model in explaining the population average association among diarrhea and the selected predictor variables. The interpretation rely on the ALR model. In ALR model parameter reflects the effect of predictors on the log odds of probability of diarrhea controlling all the other predictors in the model. Then, the odds ratio of variable can computed as (OR= ${e}^{\beta_j}$). The analysis under ALR suggests that child age is significantly associated to diarrhea disease and it was revealed that children who are between age group 0–11 months had exp.(0.5529) =1.738 times higher odds of had diarrhea than those whose age group between 24 and 59 months. Children whose age group between 12 and 23 months is exp.(0.8402) =2.32 time more odds of had diarrhea than whose age group between 24 and 59 months. This indicates that child diarrhea increased by 74% for children age group between 0 and 11 months when compared to children whose age group between 24 and 59 months. Furthermore, child diarrhea increased by 132% for children age group between 12 and 23 months as compared to children whose age group between 24 and 59 months.

From Table 2, it was indicated that a statistically significant relation between sex of child and diarrheal disease. The odds that female children had diarrheal disease is exp.(− 0.1332) = 0.875 times lower compared to male children with a diarrheal disease. Furthermore, the analysis suggests that anemia is significantly related to children diarrheal disease (*p*-value = 0.0369). This means that estimated odds of had diarrhea for anemic children is 74% more than the estimated odds for those with non-anemic children. With respect to mothers paid employment effect, it was indicated that mothers paid employment is significantly related to child diarrheal disease. This indicates that, the estimated odds of diarrhea for children belong to mothers unemployed was exp.(− 0.1979) = 0.82 times lower than the estimated odds for mothers paid employment.

In addition, it was revealed that, the estimated odds of had diarrhea for children from regions of Afar, Amhara, Dire Dawa, Gambela, Oromia, SNNPR and Tigray are respectively 75, 114, 32, 115, 107, 67, 99 and 86% more than the estimated odds of children from the central region (Addis Ababa). Furthermore, Table 2 presents that the estimated constant log odds ratios, which provides information about association between households within the same cluster. The estimated pairwise odds ratio relating two responses from the different households was found to have a small negative value (− 0.0530), this underlining the weak association in diarrheal disease between households.

#### Fever

For fever as an outcome variable, the model with child age, mother marital status, anemia level, breastfeeding status, husband education level, mother work status and region as predictor were found to be the best-fit model with the smallest QIC value. The QIC values 6996.7587 and 6996.6577 for GEE and ALR models respectively, with parameter estimates, their corresponding empirically corrected standard errors and *p*-values presented in Table 3. The QIC for GEE and ALR are almost the same. However, empirically corrected standard errors under ALR are somewhat less than those of GEE and the small differences in parameter estimates attributed to the fact that ALR considers the association unlike the GEE treat the association as a nuisance parameter. Consequently, ALR model was the better model in explaining the population average association among fever and the selected predictor variables. From Table 3, the analysis under ALR specifies that child age is significantly related to fever disease and it was observed that children who were between age group 0–11 months had exp.(0.4446) =1.556 times higher odds of had fever than those whose age group 24–59 months. Children whose age group between 12 and 23 months had exp.(0.6386) = 1.994 time more odds of children had fever than whose age group between 24 and 59 months. This indicates child fever increased by 56% for children age group 0-11 months when compared to children with had no fever. Mother marital status is the other important covariate that has a statistically significant relation with child fever disease. The odds of child from mother never in union is exp.(1.1817) = 3.289 times higher compared to child from married mother. It was observed that child from separated mother had exp.(0.8494) = 2.338 times more odds of had fever than child from married mother. This indicates that child from separated and never in union mother are associated with fever disease. With respect to husband education level, it was indicated that husband education level has statistically significantly relation with child fever disease. Higher education level of the husband was associated with 0.747 times less odds of fever in a child compared to husbands with a lower education (25% lower odds). Furthermore, the analysis suggests that breast-feeding status is associated to children fever disease. This means that estimated odds of children had fever for breast-feeding is 80% less than the estimated odds for those with not currently breastfeeding children. As it can be shown from Table 3, mothers work status is significantly related to child fever disease. This indicates that, the estimated odds of fever for children belong to unemployed mother was exp.(− 0.2352) = 0.79 times lower than the estimated odds for mother paid employment. Furthermore, region statistically related to children fever disease. The estimated odds of had fever for children from Benishangul, Somali and Tigray regions are respectively 46, 64 and 86% less than the estimated odds of children from the central region (Addis Ababa) except for Tigray region. Table 3 presents that the estimated constant log odds ratios, which provides information about association between households within the same cluster. The estimated pairwise odds ratio relating two responses from the different households was found to have a small negative association (− 0.0204), this indicates weak association between children in different households in terms of fever disease.

**Table 3:** Parameter estimates and empirical standard errors of GEE and ALR models for Fever

Marginal Model	GEE			ALR	
Effect	par	Est(s.e)	*p*-value	Est(s.e)	*P*-value
Intercept	*β*_0_	−2.719(0.4666)	<.0001	−2.7160(0.4663)	<.0001
Current age of child (24–59 months = ref)					
0–11 months	*β*_1_	0.4446(0.0898)	<.0001	0.4446(0.0898)	<.0001
12–23 months	*β*_2_	0.6384(0.0794)	<.0001	0.6386(0.0794)	<.0001
Marital status					
Married	*β*_3_	0.5706(0.3758)	0.1289	0.5694(0.3756)	0.1295
Never in union	*β*_4_	1.1839(0.4765)	0.0130	1.1817(0.4764)	0.0131
Separated	*β*_5_	0.8503(0.3988)	0.0330	0.8494(0.3987)	0.0331
Anemia level (Not anemic = ref.)					
Anemic	*β*_6_	0.3780(0.2415)	0.0175	0.3760(0.2411)	0.1188
Breastfeeding status (no = ref)					
Yes		−0.2214(0.0717)	0.0020	− 0.2215 (0.0717)	0.002
Husband education level (No education = ref)					
Higher	*β*_7_	−0.2902(0.1428)	0.0421	−0.2904(0.1428)	0.0420
Primary	*β*_8_	0.2457(0.0729)	0.0007	0.2459(0.0729)	0.0007
Secondary	*β*_9_	0.1076(0.1077)	0.3181	0.1075(0.1077)	0.3182
Currently working (Yes = ref)					
No	*β*_10_	−0.2536(0.0702)	0.0003	−0.2539 (0.0702)	0.0003
Region (Addis Ababa = ref)					
Afar	*β*_11_	0.2352(0.1833)	0.1993	0.2352(0.1833)	0.1993
Amhara	*β*_12_	−0.0868(0.1898)	0.6474	−0.0872(0.1898)	0.6460
Benishangul	*β*_13_	−0.7778(0.2109)	0.0002	−0.7791(0.2110)	0.0002
Dire Dawa	*β*_14_	−0.1015(0.2106)	0.6298	−0.1013(0.2106)	0.6304
Gambela	*β*_15_	0.1732(0.1914)	0.3655	0.1730(0.1913)	0.3660
Harari	*β*_16_	−0.3806(0.2157)	0.0776	−0.3816(0.2157)	0.0770
Oromia	*β*_17_	0.0417(0.1574)	0.7909	0.0416(0.1574)	0.7917
SNNPR	*β*_18_	0.0175(0.1681)	0.9172	0.0171(0.1681)	0.9190
Somali	*β*_19_	−0.4475(0.1849)	0.0155	−0.4473(0.1849)	0.0156
Tigray	*β*_20_	0.5627(0.1687)	0.001	0.5629(0.1687)	0.0008
Correlation	ρ	−0.002804291			
Alpha	α			−0.0204(0.0278)	0.0046
QIC		6996.7587		6996.6577	

## Discussion

This study utilized 2016 Ethiopian Demographic and Health Survey data to identify the risk factors associated with diarrhea and fever among children of under-five in Ethiopia. These indicators might be differently distributed within as well as across households, since each indicates a different mechanism by which such morbidity are acquired. The marginal models were employed to account for clustering. The study found an overall incidence of diarrhea and fever 12 and 14% respectively. For diarrhea as a response, alternating logistic regression analysis revealed that the covariates: male children, 0–11 months aged children, 12–23 months aged children, anemic children, husband with a lower education, mothers paid employment, Afar, Amhara, Dire Dawa, Gambela, Oromia, SNNPR and Tigray regions were significantly associated with higher odds of diarrhea. For fever as an outcome variable, 0–11 months aged children, 12–23 months aged children, children of never in union mother, children of separated mother, non-breastfed children, husband with a lower education, mothers paid employment, Benishangul, Somali and Tigray were significantly associated with higher odds of fever. The result shows that the age of child positively associated with the child morbidity. Children whose age group between 12 and 23 months are more probable to had diarrhea and fever morbidity than whose age group between 24 and 59 months. Similarly, children whose age group between 0 and 11 months are more at high risk of diarrhea and fever morbidity compared to the reference group. This finding is in line with that of [3, 6, 24]. In addition, the results show that risk of diarrhea is lower among female children than among male children. This finding is consistent with that of [3, 11, 24]. This may be due to biologic reasons and gender discrimination. Children caught by anemia are at higher risk of diarrhea and fever morbidity as compared to non-anemic children. This result is in agreement with previous findings such as [24, 25] among others.

Furthermore, the study found that the husband education level is an important socioeconomic factor that affects the health status of children in Ethiopia. Children from lower educated father are more at high risk of diarrhea morbidity and fever morbidity than a father at least who attended primary school. The result suggests that children from mother who are currently working are more at high risk of diarrhea morbidity and fever morbidity than children from mother who are currently not working. This result is in agreement with previous findings such as [3].

Lastly, the results indicate that children from some of regions like Amhara, Afar, Dire Dawa, Gambela, Oromia, SNNPR and Tigray are at higher risk of diarrheal morbidity compared to children from Addis Ababa. Likewise, children from Tigray region are at higher risk of fever morbidity. The finding is in line with that of [6, 26].

## Conclusions

In this paper, we studied socioeconomic, demographic, household and spatial determinants of children morbidity measured through prevalence of diarrheal and fever using Ethiopian Demographic and health Survey data. Our analyses show that marginal models are needed to adequately account for the correlation between responses of interest in subjects from the same cluster to identify the risk factors of childhood diarrhea and fever. With a traditional logistic regression model we cannot detect pairwise association of two observations in the same cluster. The alternating logistic regression analysis shows that the male children, 0–11 months aged children, 12–23 months aged children, anemic children, husband with a lower education, mothers paid employment, non-breastfed children, Amhara region, Afar region, Dire Dawa, Benishangul region, Gambela region, Oromia region, SNNPR region, Somali region and Tigray region were significantly associated with higher odds of morbidity in Ethiopia. The findings of this study have valuable implications to support strategies and interventions to address diarrhea and fever in children aged five or younger. For further study, we will focus on the geographic and socioeconomic determinants of childhood morbidity in Ethiopia.

## Data Availability

The data can be accessed from http: //www.dhsprogram.com/ by registering and requesting the datasets.

## Abbreviations

ALR: Alternating Logistic Regression
DHS: Demographic and health survey
EA: Enumeration area
EDHS: Ethiopia demographic and health survey
GEE: Generalized Estimating Equation
QIC: Qausi information criteria, first-order autoregressive, AR (1)
SNNP: Southern Nations Nationalities and Peoples Region
